# False positive finding from malignancy-like lesions on FDG PET/CT: case report of tuberculosis patients

**DOI:** 10.1186/s12880-020-00427-w

**Published:** 2020-03-05

**Authors:** Febby Hutomo, Ryan Yudistiro, Ivana Dewi Mulyanto, Hendra Budiawan

**Affiliations:** 1 Department of Nuclear Medicine, Mochtar Riady Comprehensive Cancer Centre Siloam Hospital, Jakarta, Indonesia; 2 Department of Nuclear Medicine, School of Medicine of Pelita Harapan University, Tangerang, Indonesia; 3 Department of Nuclear Medicine and Molecular Imaging, School of Medicine of Padjadjaran University, Dr Hasan Sadikin Hospital, Bandung, Indonesia

**Keywords:** Tuberculosis, Malignancy, FDG, PET/CT

## Abstract

**Background:**

The F-18 fluorodeoxyglucose positron emission/computed tomography (FDG PET/CT) has become an established diagnostic imaging for malignancy. However, there are other diseases that can also be identified with FDG, some of them are infections such as tuberculosis.

**Case presentation:**

In this case report, two patients showed multiple hypermetabolic tuberculosis lesions on FDG PET/CT, with one of the patients having history of malignancy. The objective of the present case report is to emphasize the need to use other differential diagnosis techniques for tuberculosis especially in tuberculosis-endemic countries when interpreting FDG PET/CT.

**Conclusion:**

By analyzing diagnostic imaging alone, there is a high chance of misinterpreting asymptomatic tuberculosis patient as having malignancy. Therefore, there is need for correlation with clinical data as well as other imaging modalities and PET/CT with more specific tracer in order to differentiate malignancy from benign disease such as tuberculosis.

## Background

The bacteria responsible for tuberculosis is *Mycobacterium tuberculosis*, which is a serious contagious pathogen in many countries. Based on the data released by the World Health Organization in 2009, one-third of the world’s population, almost 2 billion people, are infected with *M. tuberculosis* [1, 2].

Tuberculosis is a major health problem in most developing countries including Indonesia. The incidence rate of tuberculosis in Indonesia was 391 per 100,000 population in 2015 and was marked as the ninth most common country with Tuberculosis [3].

*M. Tuberculosis* usually affects the lung but to some extent may also involve other susceptible extra-pulmonary organs. Also, there is a challenge with the identifying the infection at the extra-pulmonary site as it is often difficult to obtain specimen for definitive diagnosis of tuberculosis [4, 5].

FDG PET/CT is a diagnostic imaging procedure which provides a unique information of cellular glucose metabolism. By using the anomalous hallmark of cancer cell in reprogramming glucose metabolism and upregulating glucose transporter, the uptake of FDG is markedly increased in cancer cells [6]. However, there are some limitations with respect to the specificity of FDG PET/CT in that it sometimes gives false negative or positive results. The false positive findings are majorly associated with high FDG uptake in infectious or inflammatory tissue [7]. Granulocytes and mononuclear cells use glucose as an energy source during their metabolic burst [8], consequently, high FDG uptake could be seen in tuberculosis. Two cases of malignancy-like lesions in tuberculosis patients were reported which resulted in false positive findings with FDG PET/CT (Table 1).

**Table 1:** Patients’ FDG PET/CT findings and the other clinical information

Patient	Age (years old)	Sex	FDG PET/CT findings	Other clinical findings
**1**	47	M	• Multiple hypermetabolic lymphadenopathies at neck and right nasopharyngeal wall. • Mild hypermetabolic ground glass opacity with thick cavitation, calcification at the apical of the left lung	• History of nasopharyngeal cancer • Normal blood test and serum CEA level within normal limit • Contrast CT scan of head and neck: no sign of relapse in the nasopharyngeal wall • Multiple lymphadenopathies at neck
**2**	58	F	• Multiple hypermetabolic lesion at the left superior bronchus wall • Mild hypermetabolic bronchial thickening at posterior part of left superior lobe • Multiple hypermetabolic lymphadenopathies at neck, mediastinum, abdominal and pelvic area. • Hypermetabolic lytic lesion at fourth lumbar • Hypermetabolic nodule at right parietooccipital region.	• History of dizziness for four months • Multiple nodules on brain CT

*FDG* fluorodeoxyglucose, *CEA* Carcinoembryonic antigen

## Case presentation

The FDG PET/CT images were acquired on a PET/CT scanner (Gemini, Phillips Healthcare, USA). All patients were subjected to fasting for over 6 h with blood sugar level below 150 mg/dL before the intravenous injection of FDG with a dose of 0.10 mCi/kgBW. The images were then acquired between 45 to 90 min after the injection. The acquisition was carried out from head to feet with arms above the head position using 700-mm field of view (FOV) and slice thickness of 10 mm. Also, three-dimensional data acquisition was performed for 3 min per bed position, followed by image reconstruction with the 3D-ordered-subsets expectation maximization method. Segmented attenuation was corrected by X-ray CT (140 kV, 120–240 mAs) to produce 128 × 128 matrix image. CT images were reconstructed using a conventional filtered back projection method.

### Case 1

A 47-year old male with nasopharyngeal cancer for 2 years complained of a stuffed nose and hearing derivation in the right ear for about 2 weeks before being subjected to FDG PET/CT. He had no history of exposure to tuberculosis patient, weight loss, cough, low-grade fever, decreased appetite, or night sweat.

The results from laboratory tests showed normal blood count and serum carcinoma embryonic antigen (CEA) level. Contrast enhanced head and neck CT scan showed no sign of relapse in the nasopharyngeal wall with multiple metastases neck lymphadenopathies.

**Fig. 1:**
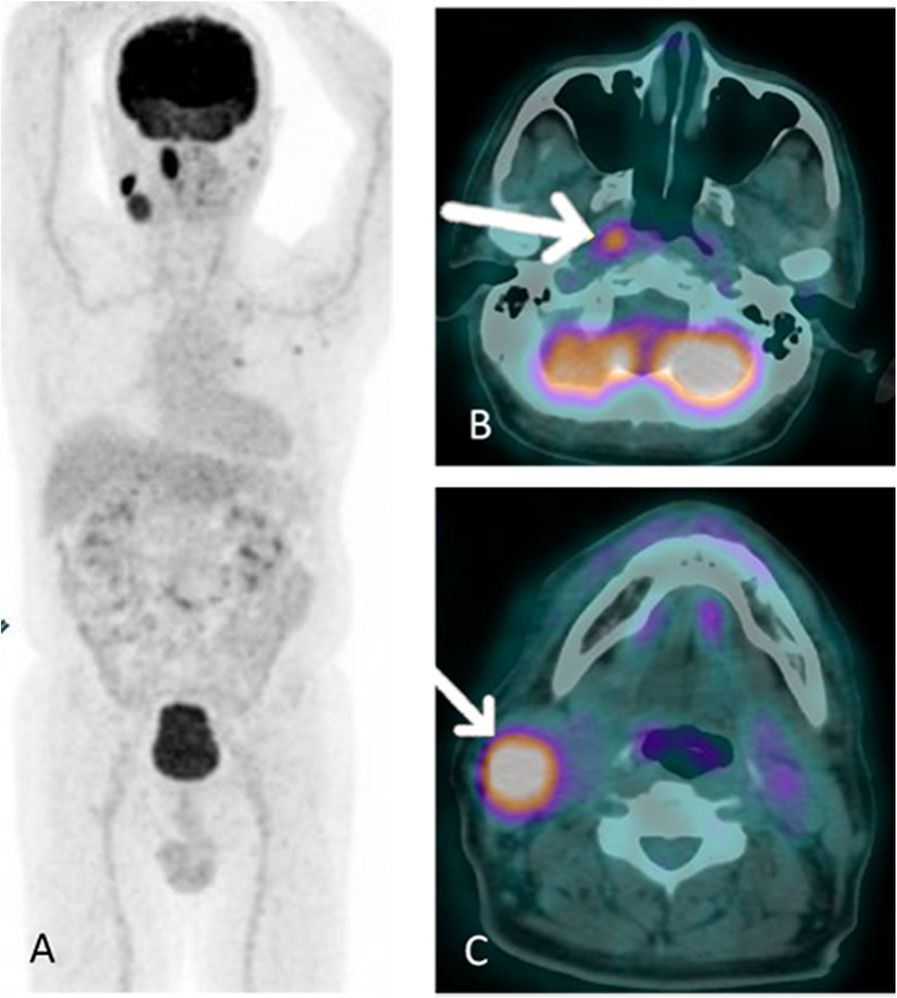
FDG PET/CT of patient number 1. Hypermetabolic lesions in several parts of head and neck. **a** MIP. **b** hypermetabolic lesion at right nasopharyngeal wall. **c** hypermetabolic enlarge lymph node at neck region

However, FDG PET/CT showed high FDG uptake by these multiple neck lymphadenopathies and the right nasopharyngeal wall (Fig. 1). The highest SUVmax of multiple neck lymphadenopathies was 11.05 that located in the right jugular superior, meanwhile SUVmax of the right nasopharyngeal wall was 4.51. Mild FDG-avid ground glass opacities with thick cavitation and calcification were also found at the apex of the left lung. Based on these FDG PET/CT findings, the biopsy was performed in the right nasopharyngeal that positive for tuberculous infection.

### Case 2

A 58-year old female presented with occasional dizziness for almost 4 months. She had history of smoking but none for tuberculosis exposure.

**Fig. 2:**
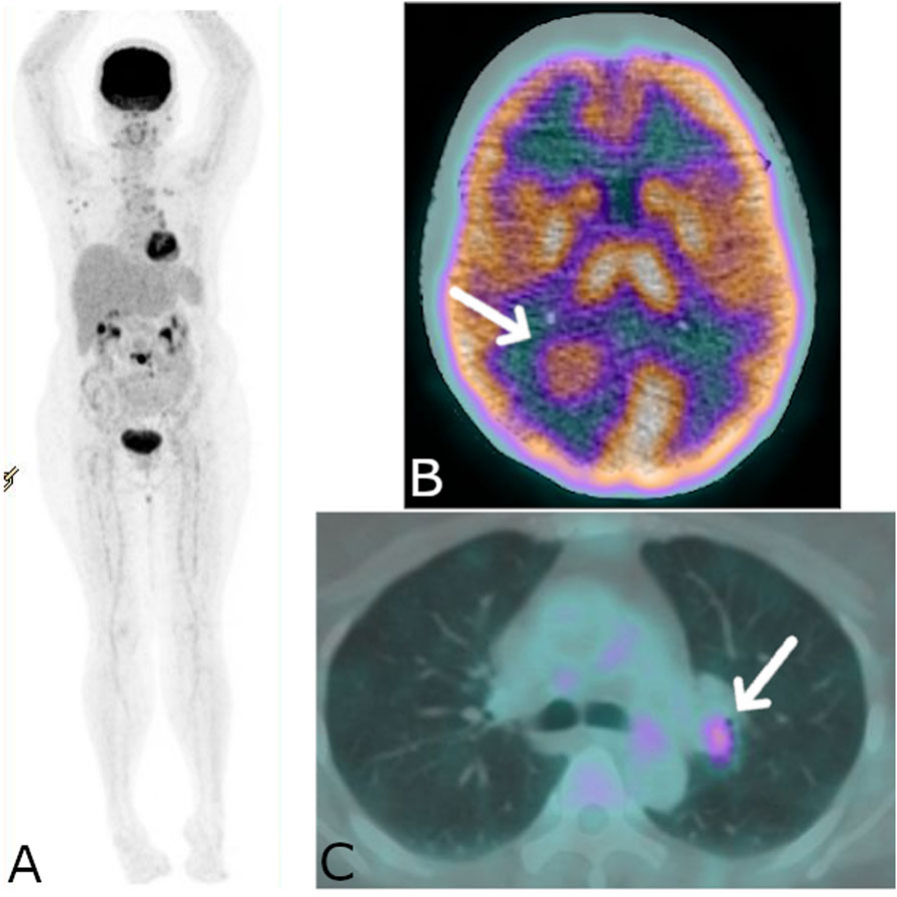
FDG PET/CT of patient number 2. **a** MIP showed multiple hypermetabolic lesions. **b** hypermetabolic nodule at brain highly suspicious for metastasis. **c** hypermetabolic lesion at left hilar region

The CT scan on the brain showed multiple metastatic nodules (Fig. 2b). Also, the FDG PET/CT showed high FDG uptake at the left superior bronchial wall (Fig. 2c) and mild FDG uptake of bronchial thickening at posterior region of the left superior lung lobe. There were also multiple high FDG uptakes at the neck, mediastinal, abdominal, and pelvic lymphadenopathies with the highest SUVmax was 4.05 that located at left hilar. High FDG uptake of lytic lesion at fourth lumbar and right parieto-occipital nodule were also seen. All these findings were interpreted as metastatic lung cancer to the lymph nodes, brain, and bone. Based on these FDG PET/CT findings, the biopsy was performed at the left hilar lymph node and positive for tuberculous infection.

All patients underwent lymph nodes biopsy and were reported as tuberculosis infections. Consequently, anti-tuberculosis drugs such as rifampicin, pyrazinamide, isoniazid, and streptomycin were administered according to the histopathology results. Also, all the patients underwent contrast enhanced CT scan three to 5 months after the initial anti-tuberculosis treatment for response evaluation. All cases showed good response to the treatment and were all considered as complete remission.

## Discussion and conclusion

There were two reported cases of malignancy-like lesions in tuberculosis patients that resulted in false positive findings with FDG PET/CT. All the lesions showed high FDG malignant-like lesions uptake and the FDG PET/CT findings misinterpreted as malignancy. However, tuberculosis was confirmed as final diagnosis by the histopathology studies.

In all these cases, the initial CT scan did not show typical characteristics finding for extrapulmonary tuberculosis, however, the FDG PET/CT showed high FDG lesions uptake which is generally viewed as malignant lesions. The extrapulmonary tuberculosis commonly develop from the pulmonary tuberculosis which subsequently spread outside the lungs through the lymphatic system. However, there are conditions whereby extrapulmonary tuberculosis develops without any evidence of pulmonary lesions [9, 10]. These two cases were found with mild FDG uptake of pulmonary lesions without any typical radiological findings for tuberculosis.

The case number one showed marked hypermetabolic lesion at right nasopharyngeal wall, suggesting nasopharyngeal cancer recurrence. This case was misinterpreted as malignancy because of the patients’ history, lesions sites, and atypical pulmonary radiologic finding lesions for tuberculosis. However, subject in case number two had no history of malignancy but showed typical metastatic distribution with hypermetabolic lesion at bronchial wall which is a suspicious sign for malignancy.

According to WHO criteria for classification, extrapulmonary tuberculosis is defined as an infection caused by M. Tuberculosis which affects tissues and organs outside the pulmonary parenchyma. The incidence rate of extrapulmonary tuberculosis is between 20 and 25% of all tuberculosis cases [11]. One of the most common extrapulmonary tuberculosis form is lymphadenopathy tuberculosis that counts for 30–40% of all extrapulmonary tuberculosis. The most common predilection site for lymphadenopathy tuberculosis is neck region (63–77%), however, it can also be found in other regions [12]. Fever and other typical systemic symptoms may not always present until the late stage of extrapulmonary tuberculosis [13].

The PET/CT study using F-18 FDG as a glucose analogue is a rapidly producing functional imaging modality which has been very beneficial in the detection of primary, recurrent, and metastatic tumour, planning, and therapy monitoring. The growth of cancer cell is an energy-related process which is supported by increased glucose metabolism. It is widely known that glucose transporter (GLUT) proteins, a membrane protein, are responsible for the transport of glucose across cellular membranes. Consequently, cancer cells have higher rates of GLUT expressions compared to normal cells. Together with this mechanism, some types of tumour is also associated with a higher rate of phosphorylation, lower rate of dephosphorylation of intracellular phosphorylated glucose, and higher activity of hexokinase.

These characteristics of cancer cell make the FDG accumulation much higher than normal cell. FDG PET/CT is known as superior but less specific for diagnostic imaging in malignancy. Through glucose transporters that are overexpressed both in cancer and inflammatory cells hence result in FDG accumulation, is not only visible in cancer cells but also in inflammatory cells such as activated macrophages, lymphocytes, and neutrophils at the site of inflammation or infection [14].

There is a similarity between the FDG uptake mechanism of both cancer and inflammatory cells. Both cells need high glucose consumption to fulfill the high energy demands. Glucose enter the cells through energy-dependent transporters. There are seven types of glucose transporter, known as GLUT-1 to GLUT-7 [11]. It was discovered in a study that a relative higher FDG uptake was observed in some of the inflammatory lesions, in which the expression level of GLUT-3 was much higher compared to GLUT-1. Also, Fu *et all* found that mRNA analysis in inflammatory cells showed GLUT-1 expression increased to 3.5 times, whereas GLUT-3 increased to 6 times, following the activation of inflammatory factors [9, 12]. The expressions of GLUT-1 and GLUT-3 in the inflammatory lesions are related to the type, quantity, and degree of activation of the inflammatory cells. Another study reported the important roles played by cytokines and growth factors in promoting the affinity of glucose transporter. Therefore, FDG uptake mechanism in inflammatory cells is almost similar compared to tumour setting though different situations [9].

Many studies have been conducted in attempting to differentiate between malignancy and inflammation in FDG PET study, including variation of protocols, characteristic, and parameter. Traditionally, a threshold for single time point using SUV max has been proposed to differentiate the two processes, however this method was proven not effective with false positive rate above 60%. Other widely accepted method is using dual-time imaging, based on varying levels of glucose-6-phosphatase activity among different tumour cell types, inflammatory and normal cells. However, this method is not dependable for several reasons including histologic type of tumours, the combination or the coexisting of chronic and acute inflammation, necrosis, hypoxia, and degree of angiogenesis. Most recent proposed method is using influx rate constant (Ki). It is reported in lung inflammatory lesions that this parameter is closely related to neutrophil activation. However, it is not applicable in daily practice and therefore requires further study in various type of tumours.

Familiarity with oncologic pattern and correlation with other modalities such as tumour marker and anatomical imaging are important in FDG PET/CT interpretation to produce more accurate assessment in clinical setting. Also, it is important to understand the pitfall of tumour markers and other clinical assessments commonly used in diagnosing malignancy. Several studies showed CEA level which might also increase in inflammatory process in the lung [15, 16]. New specific radiopharmaceutical for tuberculosis, such as Tc-99 m-ethambutol might also be helpful in these setting [17, 18].

Conclusively, two cases of malignancy-like lesions in tuberculosis patients were reported but resulted in false positive findings in FDG PET/CT. By analyzing only diagnostic imaging, asymptomatic tuberculosis patient can be easily misinterpreted as having malignancy. There is need for correlation with clinical data, as well as other imaging modalities and PET/CT with more specific tracer, in order to be able to differentiate malignancy from benign disease such as tuberculosis.

## Data Availability

The datasets generated and/or analysed during the current study are de-identified and not publicly available considering the fact that not all the patients consented to it for publication but are available from the corresponding author on reasonable request.

## Abbreviations

CEA: Carcinoma Embryonic Antigen
CT: Computed tomography
FDG: ^18^F-fluorodeoxyglucose
GLUT: Glucose transporter
PET: Positron emission tomography
